# Artemisinin Resistance Outside of Southeast Asia

**DOI:** 10.4269/ajtmh.18-0845

**Published:** 2018-11-12

**Authors:** Philip J. Rosenthal

**Affiliations:** Department of Medicine, University of California, San Francisco, San Francisco, California

Drug resistance has challenged malaria control since the development of chloroquine resistance in the 1950s. Over about the last decade, resistance of *Plasmodium falciparum* to artemisinins, presenting as delayed clearance after therapy with an artemisinin or artemisinin-based combination therapy (ACT), emerged in Southeast Asia.^1^ Potential spread of artemisinin resistance to other areas is of great concern because if resistance migrates to areas with very high malaria burdens, in particular sub-Saharan Africa, the consequences may be devastating.

Artemisinin resistance in Southeast Asia is now well-characterized, recognized clinically by delays in clearance of parasites after treatment with artemisinin-based regimens, molecularly by nonsynonymous mutations in the propeller domain of the *K13* gene (*K13PD*), and parasitologically by decreased clearance in the ring survival assay.^1^ By all these measures, artemisinin resistance is prevalent in the Greater Mekong Subregion, which extends from the epicenter in Cambodia to parts of Vietnam, Laos, Thailand, Myanmar, and China.^2^ The easiest means of surveillance for artemisinin resistance is sequencing to assess *K13PD* mutations, and broad characterization of *K13PD* sequences has been carried out in recent years.^3,4^ *K13PD* mutations have been seen in parasites from many areas, but it appears that only a subset of these impact on drug sensitivity. One mutation, *C580Y*, is now the dominant *K13PD* mutation mediating artemisinin resistance in much of the Greater Mekong Subregion.^5^ However, multiple other mutations are also associated with delayed clearance after therapy; a recent pooled analysis extended the number of *K13PD* mutations associated with delayed clearance to 20.^6^ With this improved understanding, and with many recent surveys of *K13PD* sequences, it is useful to review our understanding of artemisinin resistance in other regions of the world.

Two new articles in the *AJTMH* offer updated insights into the status of artemisinin resistance in Brazil^7^ and India.^8^ Other recent reports offer information on Africa and other areas. Overall, there remains scanty evidence for artemisinin resistance outside of Southeast Asia. For the moment, we can breathe sighs of relief. Or can we? As yet unpublished reports suggest the migration of artemisinin resistance to eastern India, and recent publications offer hints of resistance elsewhere. Whether or not resistance has already spread beyond Southeast Asia, past experience with other antimalarials suggests that artemisinin resistance is likely to spread around the world over time. But what do recent articles show about the present situation?

South America is an area of relatively low malaria transmission intensity. Low transmission areas may be particularly prone to the emergence of drug resistance, due to low antimalarial immunity in the population and low incidence of polyclonal infections, both facilitating the establishment of relatively unfit drug-resistant infections. Indeed, resistance to most available antimalarials first emerged in Southeast Asia and/or South America, but not Africa. A new report in the *AJTMH* identified complete absence of *K13PD* mutations among 152 *P. falciparum* isolates collected from the Amazon region of Brazil, mostly before the introduction of ACTs, but including 34 isolates collected after the change in treatment policy, mostly collected in 2010–2011. These results are consistent with other recent reports from Brazil, including identification of only one *K13PD* mutation among 237 *P. falciparum* isolates collected in Amazonas state in 2014,^3^ no *K13PD* mutations among 162 samples collected in Acre state in 2010–2013,^9^ no *K13PD* mutations among 69 isolates collected in four different regions in 2010–2017,^10^ and no *K13PD* mutations among 31 isolates collected in Acre state up to 2005.^4^ Elsewhere in South America, none of the 163 *P. falciparum* isolates collected from patients with uncomplicated malaria in Colombia in 2014–2015^11^ and none of 40 isolates collected in Suriname in 2013–2014 had *K13PD* mutations.^12^ By contrast, *K13PD* mutations were identified in Guyana, with five of 98 *P. falciparum* isolates collected in 2010 containing the *C580Y* mutation that is commonly associated with resistance in Southeast Asia; molecular data suggested emergence independent from that of Asian parasites.^13^

India has varied malaria transmission intensity, but overall one of the highest malaria burdens in the world. Its location suggests that it may serve as a portal for the spread of artemisinin resistance from Southeast Asia. However, *K13PD* mutations have remained uncommon in most studies of *P. falciparum* from India. A new report in the *AJTMH* showed that none of 112 isolates collected in Mangaluru, in southwestern India, in 2015, contained *K13PD* mutations.^8^ Other studies from India found *K13PD* mutations in none of 51 isolates collected in Kolkata in 2014^14^; three of 186 isolates collected from four districts in 2014–2015; and two of 254 isolates collected in northeastern India in 2014–2015.^15^ One study showed quite different results, with 50 of 135 isolates with two *K13PD* mutations, although these mutations were not associated with delayed clearance after therapy with an ACT^16^; the finding of more than one propeller domain mutation in a single isolate is unusual, and the reason for discrepancies in results between this report and others is unknown. Considering *P. falciparum* from other countries in Asia outside of the Greater Mekong Subregion, *K13PD* mutations were found in one of 253 isolates collected in seven districts of Bangladesh in 2009–2013^17^; none of 61 isolates collected in Papua, Indonesia, in 2015–2016^18^; none of 50 isolates collected in Malaysia in 2011 and 2014^19^; and two of 60 isolates collected in Afghanistan in 2012–2014.^20^

Extensive data are available to consider the spread of artemisinin resistance to Africa. Clinical trials have shown consistently strong efficacy of ACTs to treat uncomplicated malaria in Africa, with clearance of parasites after therapy almost always seen within 2–3 days.^2,21^ The in vitro ring survival assay has also shown rapid clearance of African parasites after incubation with artemisinins.^22^ Sequencing studies have shown low but varied prevalence of *K13PD* mutations, with prevalence < 5% at nearly all sites, but identification of a large number of different mutations.^3,23,24^ Most of these mutations, including *A578S*, the most common *K13PD* mutation identified in Africa, have not been associated with delayed clearance in Asia.^6^ However, in Uganda, among 78 children diagnosed with severe malaria, three had isolates with the *A578S K13PD* mutation, and parasite clearance was delayed in these children compared with that in the full cohort.^25^ Another mutation, *A675V*, which has been associated with delayed clearance in Asia,^6^ was seen in one isolate from northern Uganda with in vitro delayed clearance.^26^ This mutation was seen in about 5% of isolates from nearby regions of northern Uganda,^27^ but its clinical relevance is unclear.

The available data lead to the following conclusions. First, artemisinin resistance, defined as delayed clearance after treatment with artemisinin-based therapies, is entrenched in the Greater Mekong Subregion. With development of resistance to some artemisinin partner drugs, in particular piperaquine, failures of ACT therapy for falciparum malaria are now common in parts of this region, an alarming development. Second, we do not see clear evidence of artemisinin resistance outside of Southeast Asia, although there are hints suggesting emergence of *P. falciparum* with characteristics of resistant parasites in some areas. Third, considering the enormous potential consequences of the spread of artemisinin resistance, continued surveillance for resistant parasites, in particular taking advantage of the ease of *K13* characterization, is warranted.
